# Ticagrelor-Induced Torsades de Pointes following Myocardial Infarction

**DOI:** 10.1155/2022/4505964

**Published:** 2022-07-21

**Authors:** Seong Hyeon Bu, Sung-Hwan Kim

**Affiliations:** ^1^Division of Cardiology, Department of Internal Medicine, Uijeongbu St. Mary's Hospital, College of Medicine, The Catholic University of Korea, Uijeongbu-si, Gyeonggi-do, Republic of Korea; ^2^Division of Cardiology, Department of Internal Medicine, Seoul St. Mary's Hospital, College of Medicine, The Catholic University of Korea, 222 Banpo-daero, Seocho-gu, Seoul, 06591 Seoul, Republic of Korea

## Abstract

We report a case of excessive QT prolongation and subsequent torsades de pointes (TdP) following the administration of ticagrelor in a 58-year-old male patient. The patient had no suspected cause of QT prolongation. After cessation of ticagrelor, QT interval was normalized and no further TdP was observed.

## 1. Introduction

Torsades de pointes (TdP) is caused by bradycardia, drugs, electrolyte imbalance, congenital long QT syndrome, and metabolic diseases [1]. TdP following myocardial infarction (MI) is rare. If polymorphic ventricular tachycardia (VT) occurs in a patient with acute MI, coronary revascularization should be prioritized to resolve ischemia [2]. We encountered a patient that presented with TdP after complete revascularization. After cessation of ticagrelor, QT interval was normalized and no further episode of TdP was observed.

## 2. Case Description

**Figure 1:**
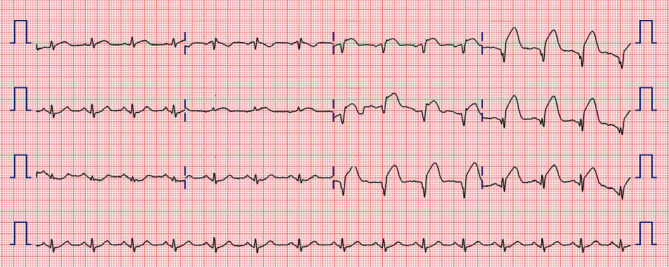
Initial ECG of a 58-year-old man in our hospital. This ECG reveals ST elevations in leads V_1_-V_6_ and reciprocal changes in leads II, III, and aVF. ECG: electrocardiogram.

**Figure 2:**
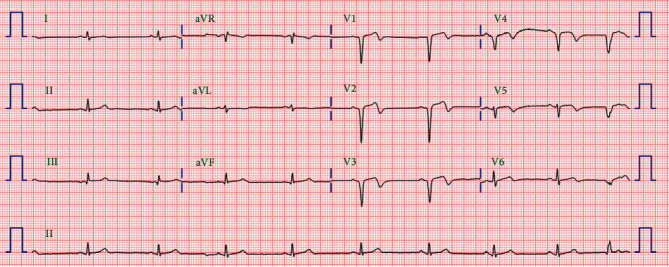
On the fourth day of hospital discharge, the ECG reveals no significant QT prolongation.

A 58-year-old man presented to the emergency room one hour after the sudden onset of squeezing chest pain. The initial electrocardiogram (ECG) revealed ST elevations in leads V_1_-V_6_ and reciprocal changes in leads II, III, and aVF (Figure 1). Emergency coronary angiography was performed, and a loading dose of 180 mg of ticagrelor, 300 mg of aspirin, and 40 mg of atorvastatin were subsequently administered to the patient. His medical history included chronic smoking. His blood pressure was 136/76 mmHg and heart rate 87 beats/min. Chest radiography showed mildly increased pulmonary vascularity. An emergent coronary angiogram (CAG) revealed an acute thrombus completely obstructing the proximal left anterior descending coronary artery (LAD), for which the stent was immediately and successfully deployed. Transthoracic echocardiography demonstrated a left ventricular ejection fraction of 0.45 to 0.49 and a regional wall motion abnormality in the LAD territory. On the fourth hospital day, he was discharged on aspirin, ticagrelor, atorvastatin, and bisoprolol. The ECG revealed no significant QT prolongation (Figure 2).

**Figure 3:**
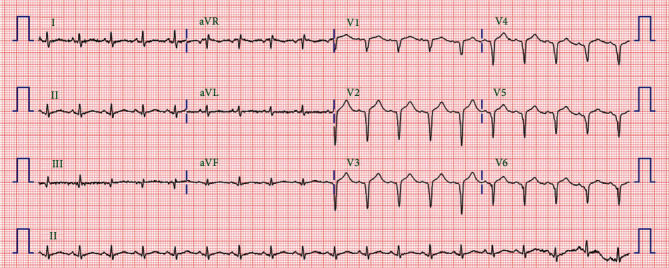
A second ECG taken after ROSC following cardiopulmonary arrest. ECG shows QT prolongation. ECG: electrocardiogram; ROSC: return of spontaneous circulation.

**Figure 4:**
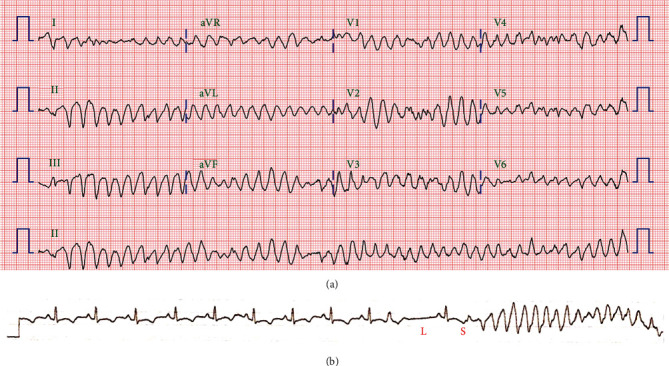
Polymorphic VT, (a) which starts in a pause-dependent manner with a long-short sequence and (b) is observed frequently after ROSC. Hence, the initial diagnosis is TdP following an MI. VT: ventricular tachycardia; ROSC: return of spontaneous circulation; TdP: torsades de pointes; MI: myocardial infarction.

**Figure 5:**
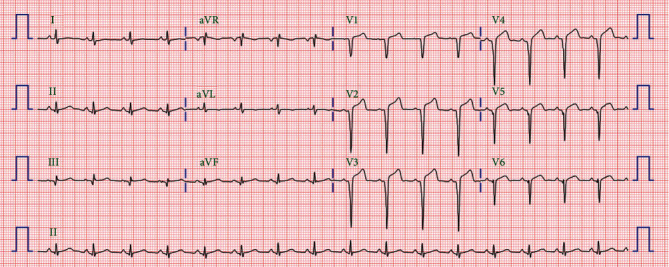
Repeat ECG following discontinuation of ticagrelor. There is shortening of the previously prolonged QT interval. ECG two days after ticagrelor discontinuation. No further cardiac events are sustained in the follow-up period. ECG: electrocardiogram.

However, on the third day following discharge, the patient was brought to the emergency room in a state of cardiac arrest. In the morning prior, he was found collapsed in the bathroom. He had ventricular fibrillation. After five cycles of defibrillation, he recovered and was admitted to the emergency room. At the time of admission, ECG showed no ST elevation, his blood pressure was 83/54 mmHg and heart rate 72 beats/min, and he was in a state of stupor. However, his blood pressure gradually improved to 98/55 mmHg and heart rate to 87 beats/min, and he was noted to be confused. CAG showed a patent stent, and optical coherence tomography showed no findings of malapposition, underexpansion, or thrombus in the culprit vessel. However, ECG showed QT prolongation (Figure 3). Polymorphic VT, which started in a pause-dependent manner with a long-short sequence, was observed frequently (Figures 4(a) and 4(b)). No electrolyte imbalances, including hypokalemia, were observed, and no sedative drugs were prescribed. Intravenous isoproterenol was used to increase the heart rate, and magnesium was administered intravenously. TdP continued for more than 6 days after readmission. We suspected ticagrelor as the cause of QT prolongation and subsequent TdP. Thus, we discontinued ticagrelor and replaced it with clopidogrel. Subsequently, the QT interval was shortened, and no further TdP occurred (Figure 5). After discharge, there were no specific findings for a follow-up period of 3 months.

## 3. Discussion

When polymorphic VT occurs after MI, three possibilities should be considered: ischemic ventricular fibrillation due to acute ischemia, polymorphic VT with QT prolongation (so-called pseudo-TdP), and true TdP with certain causes of QT prolongation. Ischemic ventricular fibrillation in the setting of acute MI generally occurs during the hyperacute phase, is related to ischemia, and is not associated with QT prolongation. It can be excluded in this case [1]. QT prolongation after MI usually appears within a day or two prior to subsequent normalization [3]. In a case series comparing 8 MI patients with TdP and 100 without TdP, the QT interval in patients without TdP increased significantly on the second day and subsequently returned to normal within the third day after MI [4]. The coupling interval and pause-dependent pattern help discriminate between polymorphic VT that occurs despite QT prolongation (pseudo-TdP) and true TdP with certain causes of QT prolongation. The coupling interval of the ectopic beat triggering the pseudo-TdP was much shorter than that of true TdP.

The present case showed QT prolongation, polymorphic VT with a pause-dependent, long-short sequence, relatively long coupling interval of triggering TdP, and no acute ischemia. These evidences clearly supported the present TdP which would have a certain cause of QT prolongation rather than acute ischemia. TdP ensued until the sixth day of rehospitalization and did not improve. Therefore, we investigated other potential causes. There was no bradyarrhythmia or electrolyte imbalance. There were no medications administered other than the ones prescribed. A previous study demonstrated that ticagrelor blocks the uptake of adenosine by red blood cells and significantly increases the concentration of adenosine in the blood [5]. There was a report that adenosine induced TdP [6]. Another case report noted an association between prolonged QT and ticagrelor [7]. Based on these literatures, we decided to discontinue ticagrelor. From the evening after the discontinuation of ticagrelor, the QT interval shortened and no further episodes of TdP were observed. The half-life of ticagrelor was known to be between 7.7 and 13.1 hours [8], suggesting that it is the cause of the event. QT prolongation associated with ticagrelor use should be considered after exclusion of important other causes like excluding ongoing cardiac ischemia, electrolyte abnormalities, and drugs.

The mechanism of QT prolongation involves several potassium channel blockades affecting the repolarization phase of myocytes [9]. One study showed that a single oral administration of 900 mg ticagrelor did not prolong the QT interval after 24 hours in healthy subjects [10], and it is not clear whether the resolution of QT prolongation in this patient was due to the replacement of ticagrelor with another antiplatelet agent or otherwise spontaneous improvement. However, it is worth considering whether ticagrelor affects the potassium channels or causes QT prolongation by other mechanisms.

In conclusion, QT prolongation following MI induced TdP in our patient. We were able to identify a temporal and causal relationship between ticagrelor discontinuation and the resolution of QT prolongation.

## Data Availability

No data were used to support this study.
